# Effects of Extrinsic Wheat Fiber Supplementation on Fecal Weight; A Randomized Controlled Trial

**DOI:** 10.3390/nu12020298

**Published:** 2020-01-22

**Authors:** Beate Brandl, Yu-Mi Lee, Andreas Dunkel, Thomas Hofmann, Hans Hauner, Thomas Skurk

**Affiliations:** 1 ZIEL—Institute for Food & Health, Technical University of Munich, Gregor-Mendel-Str. 2, 85354 Freising, Germany; beate.brandl@tum.de (B.B.); hans.hauner@tum.de (H.H.); 2 Else Kröner-Fresenius-Center of Nutritional Medicine, Technical University of Munich, Gregor-Mendel-Str. 2, 85354 Freising, Germany; Yu-Mi.Lee@eitfood.eu; 3 Leibniz-Institute for Food Systems Biology at the Technical University of Munich, Lise-Meitner-Straße 34, 85354 Freising, Germany; a.dunkel.leibniz-lsb@tum.de (A.D.); thomas.hofmann@tum.de (T.H.); 4 Chair of Food Chemistry and Molecular Sensory Science, Technical University of Munich, Lise-Meitner-Straße 34, 85354 Freising, Germany; 5 Institute of Nutritional Medicine, Klinikum rechts der Isar, Technical University of Munich, Georg-Brauchle-Ring 62, 80922 Munich Germany

**Keywords:** wheat fiber, fecal bulk, fecal wet weight, fecal dry weight, gut microbiota

## Abstract

Higher fiber intake may confer beneficial effects on health. Our objective was to investigate the impact of 10 g extrinsic wheat fiber on fecal bulk. Therefore, we performed two randomized intervention studies in which we provided extrinsic wheat fiber-enriched products or appropriate control products for five days together with normal diet. In one trial, 10 participants received fiber-enriched food products, whereas in the second study, 19 participants supplemented their daily diet with fiber-enriched drinks. The main outcome variable of this intervention was fecal bulk. Other outcomes were gut microbiota composition, short chain fatty acids in feces, and stool consistency and frequency. Fecal wet weight was significantly increased (*p* < 0.02) with extrinsic wheat fiber-enriched foods. In contrast, ingestion of extrinsic wheat fiber in the form of drinks did not significantly change fecal wet weight. In both groups, fecal dry weight was not altered upon extrinsic wheat fiber supplementation. However, the intake of fiber-enriched foods resulted in higher acetic acid levels in feces compared to fiber-enriched drinks. Regarding gut microbiota profiles, extrinsic wheat fiber-enriched food products were not associated with substantial alterations. In conclusion, 10 g extrinsic wheat fiber added to a normal diet increased fecal bulk if administered in a solid food matrix, but not if applied in the form of drinks. DRKS, DRKS00015792.Registered 30 October 2018.

## 1. Introduction

Dietary fibers have been shown to exhibit different physiological effects and to provide a plethora of positive health outcomes, such as satiation [1,2], and regulation of digestion [3] and cardiovascular disease [4,5], thereby reducing the risk of numerous diet-related diseases such as diabetes mellitus type 2 [6,7]. Regarding glucose metabolism, besides insoluble resistant starch [8] and soluble dietary fibers [9], the consumption of highly purified insoluble dietary fibers from wheat was associated with reducing the post-prandial glucose response [10]. Other positive effects can be attributed to an increased stool volume [11,12] and a reduced transit time [11]. In addition to health effects related to glucose metabolism, beneficial effects of fibers on fecal bulk were shown in the systematic review of Vries et al. [13]. Nowadays, predominantly insoluble fibers are considered to increase the stool volume and enhance the frequency of bowel movements. Moreover, studies indicate that dietary fibers are able to bind bile acids, which prevents reabsorption in the ileum and increases bile acid synthesis. As a result, a cholesterol-lowering effect with the consumption of dietary fibers was described [14].

In previous intervention studies, an increase in the amount of stool volume upon addition of dietary fibers in the form of sugar beet, barley, or oatmeal fibers to the diet has already been shown [15,16]. The aim of the present study was to investigate the impact of supplementation with 10 g extrinsic wheat fiber, equally distributed across the main meals, on fecal bulk under a regular diet. On the one hand, we applied the fibers in the form of baked/cooked solid foods and, as a pilot experimentation, we additionally administered them as a flavored drink to address the potential matrix effects of those products.

## 2. Materials and Methods

All procedures were approved by the ethics committee of the Faculty of Medicine of the Technical University of Munich in Germany (approval code 425/18S-KK) and were in accordance with the Declaration of Helsinki. Written informed consent was obtained from all participants before inclusion.

### 2.1. Study Participants

For this trial, in total, 20 adults (12 females, 8 males) aged 18–30 years were recruited. The recruitment took place between October 2018 and March 2019 on the campus of the Technical University of Munich in Freising, Germany. The participants’ eligibility was assessed with a detailed screening questionnaire. Exclusion criteria were: BMI > 30 kg/m^2^, smoking, intestinal diseases, acute infections, and antibiotic treatment <6 months ago. After enrollment, participants were randomly allocated. Allocation concealment was performed with SNOSE (sequentially numbered, opaque sealed envelopes). Treatments were encoded with M or Y for solid foods and Z or N for the drinks. Encoding was performed by an employee of J. Rettenmaier & Söhne GmbH + Co KG (JRS, Rosenberg, Germany) not involved in the study conduct.

### 2.2. Study Product

The fiber product (VITACEL^®^, J. Rettenmaier & Söhne GmbH + Co KG, Rosenberg, Germany) is a dietary fiber concentrate extracted from the wheat plant. It is exclusively composed from 66% cellulose, 28% hemicellulose, and virtually no lignines or beta-glucan. Also, no fructanes that are classified as FODMAP (fermentable oligo-, di- and monosaccharides and polyols) can be detected in the product. Those compounds are considered to cause digestive discomfort in susceptible people. This depletion is achieved by basic (NaOH) digestion, which removes lignins, and aqueous extraction of the wheat plant, which removes fructanes, beta-glucan, and proteins.

### 2.3. Study Design

**Figure 1:**
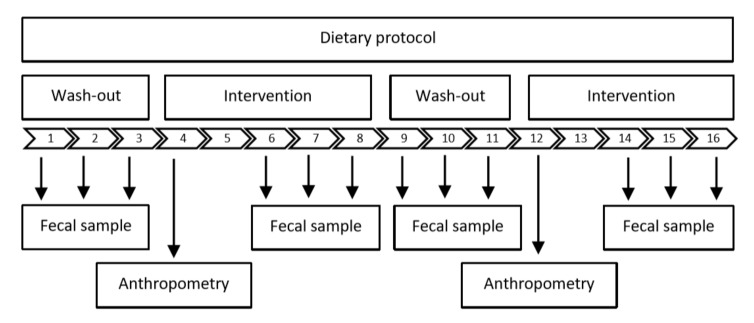
Study design of the food and beverage studies. Timeline and examinations.

Figure 1 depicts the study design of the two interventions. Each study was randomized, placebo-controlled, double-blind, and crossover. Participants were invited to either receive solid foods supplemented with fibers or not (intervention 1), or fiber supplemented or placebo drinks (intervention 2). Each test conditions lasted five-days and participants were informed to keep their usual eating habits. Regarding intervention 1, all participants received, in addition to their usual diet, the extra dietary fiber in form of foods such as bread, pizza, curry sausage, meatballs, and muffins. Participants could choose the product they wanted to consume (Supplementary Materials, Figure S1). In order to ensure that the dietary fiber intake is close to everyday life, the intended supplementation with 10 g fibers was portioned into 3 × 3.34 g servings of extrinsic wheat fiber per day. During the control phases, participants received the same products without added dietary fibers. It is noteworthy that the fiber-enriched products and control products had a similar taste and appearance. The second trial was defined as pilot study, where volunteers received fibers (or placebo) in form of a powder that had to be dissolved in 300 mL water. To ensure compliance, participants received all study foods and drinks at the Core Facility Human Studies of the ZIEL- Institute for Food & Health, Freising, Germany. Participants were monitored to ensure that they completely consumed the test products. Between the intervention phases, there was a wash-out period of 3 days. During the wash-out phase and during the last three days of each intervention, all participants’ fresh feces were weighed, whereby the last fecal sample was additionally freeze dried. Furthermore, anthropometry and body composition were analyzed in every intervention phase to ensure stable conditions. In addition, gut microbiota composition, stool consistency categorized via the Bristol Stool Chart [17], and stool frequency were determined. Finally, during the whole study, participants were asked to record their dietary intake in standard forms and complete an analog scale regarding product liking.

### 2.4. Anthropometry and Body Composition

Measurements were performed in a standardized manner after an overnight fasting period. Body height was measured using a stadiometer (Seca, Hamburg, Germany). Waist was measured with a soft tape midway between the lowest rib and the iliac crest. Hip circumference was measured at the widest part of the gluteal region. Body weight and body composition was assessed by using Seca mBCA 515 (Seca GmbH & Co KG, Hamburg, Germany).

### 2.5. Fecal Samples

Fecal samples were collected into Fecotainer (AT Medical BV, Enschede, Netherlands) before and after intervention on three consecutive days. In addition, fecal samples were weighed after freeze drying (BenchTop Pro with Omnitronics, SP Scientific, NY, USA). Moreover, participants categorized their fecal sample according to the Bristol Stool Form Scale [17]. Before and after every intervention, the participants collected stool samples into two tubes according to a standardized procedure, whereby one tube contained 8 mL DNA stabilization buffer (Stratec Molecular GmbH, Berlin, Germany). Participants were instructed to bring the samples to the study center within 6 hours, where they were subsequently stored at −80 °C.

### 2.6. Dietary Protocols

The study participants were instructed to record their food consumption during the whole study period. The supplemented 10 g extrinsic wheat fiber was specially taken into account in the calculations. The energy content and macronutrient composition of the diets were calculated using the OptiDiet Plus software (Version 5.1.2.046, GOE mbH, Linden, Germany).

### 2.7. Quantitation of Short-Chain Fatty Acids (SCFAs)

SCFA measurement was performed by LC-MS/MS after 3-nitrophenylhydrazine derivatization using a recently reported method with some modifications [18]. Detailed information about the measurement is shown in the supplemental (Supplementary Materials).

### 2.8. High-Throughput 16S rRNA Gene Sequencing

Raw sequence reads were processed based on the UPRASE [19] approach using IMNGS [20]. Operational taxonomic units (OTUs) were clustered at a threshold of 97% sequence similarity. To improve the quality of the data and increase the number of false positives, OTUs with a relative abundance of <0.25% were considered as artefacts and, thus, as sequencing contaminates and were considered during the analysis.

### 2.9. Data Analysis and Statistics

Data were analyzed in the R programming environment. Results are presented as mean ± SD and *p*-values < 0.05 were regarded as statistically significant. Shapiro–Wilk (normality) test was used to test normal distribution of data. According to distribution, either the paired *t*-test or Wilcoxon-signed ranked test was applied to assess differences between control diet and extrinsic wheat fiber-enriched diet. The Mann–Whitney *U* test was used to test for the significance between the two intervention studies or differences between males and females. For downstream analysis of OTUs, the Rhea pipeline was used [21]. Regarding the intervention 1 (food) sample, size estimation was performed for the fecal wet weight. Power calculations were performed for a two-way repeated measures analysis of variance (ANOVA) using the software R. Expected values of means and standard deviations of the outcome variable were obtained from the literature and used to simulate 10,000 experimental data sets with various sample sizes ranging from 5 to 50 individuals per group. Those data sets were each statistically analyzed using the R package lme4. The percentage of retrieved significant coefficients was related to the sample size and indicated the expected power of the experiment. Intervention 2 with beverages was carried out as a pilot to measure the effect size of the habitual intake during meal times.

## 3. Results

### 3.1. Subject Characteristics

In total, 48 interested volunteers were screened according to our inclusion and exclusion criteria. Finally, in total, 20 participants (24.3 ± 3.6 years, 22.2 ± 2.0 kg/m^2^) were included in the study. Comparing both groups, participants’ mean age, body mass index, and body composition did not vary significantly. Within each group, all study conditions did not significantly alter anthropometric and body composition parameters (Supplementary Materials, Table S1).

### 3.2. Supplementation with Extrinsic Wheat Fiber Increased Fecal Bulk

Table 1 summarizes the fecal wet weight and fecal dry weight as well as stool consistency and stool frequency between the groups. With a mean difference of 64.7 ± 49.9 g/day, fecal wet weight increased significantly for the extrinsic wheat fiber-enriched diet in form of solid food (*p* = 0.02). In females (*n* = 5), fecal wet weight increased from 42.3 ± 28.1 g/day to 175.1 ± 64.5 g/day by eating a supplemented extrinsic wheat fiber-enriched diet in the form of solid food. Fecal wet weight in male participants (*n* = 5) increased by 87.1 ± 63.1 g/day resulting in a mean fecal wet weight of 243.1 ± 102.1 g/day at the end of the intervention. It is noteworthy that an increase of fecal bulk could not be detected by the fiber-enrichment of drinks, despite a having higher number of participants. In this group, the fecal wet weight in females (*n* = 12) increased from 152.8 ± 55.1 g/day to 163.9 ± 66.2 g/day, and in the male volunteers (*n* = 7) from 199.3 ± 60.6 g/day to 202.8 ± 129.0 g/day by the addition of extrinsic wheat fibers to the diet. Moreover, in both groups (food and drink), fecal dry weight and stool frequency did not change. Furthermore, participants had a mean stool consistency between 3 and 4 in both study groups, which is generally considered to be the ‘normal’ stool form. No gender differences could be detected in both intervention studies regarding consistency.

### 3.3. Extrinsic Wheat Fiber-Enriched Foods Do Not Alter SFCA Levels vs. Control Diets

Comparison between the control and the extrinsic wheat fiber-enriched diet did not reveal significant changes in SCFA concentrations between the two conditions (Table 2). However, comparing the SCFAs between both fiber treatments (food and drink), acetic acid (*p* = 0.04) was significantly different with higher levels in the solid food group compared to drinks. Regarding the control diets, propionic acid was significantly increased in the control diet in the form of drinks (1087.6 ± 469.9 nmol/mL) compared to solid food (736.4 ± 259.3 nmol/mL, *p* = 0.04).

### 3.4. Extrinsic Wheat Fiber Altered Microbiota Composition

**Figure 2:**
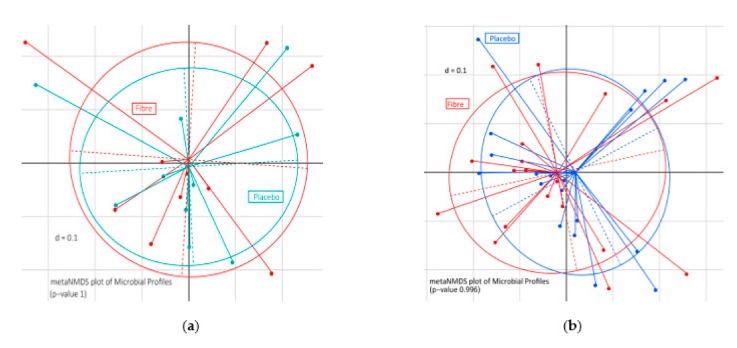
Fecal microbiota analysis by 16 S rRNA gene amplicon analysis. (**a**) Extrinsic wheat fiber-enriched foods, and (**b**) extrinsic wheat fiber-enriched drink. metaNMDS is meta non-parametric, multidimensional scaling plot of phylogenetic distances based on generalized UniFrac (beta-diversity).

As expected, beta-diversity revealed marked inter-individual differences without significant clustering according to the intervention and placebo (Figure 2a,b). The dominant gut microbial phyla were *Firmicutes*, *Bacteroidetes*, *Actinobacteria*, *Proteobacteria*, and *Verrucomicrobia*. The phyla *Firmicutes* represented, in both intervention studies, between 52–65% of gut microbiota, and the phyla *Bacteroidetes* represented 31–43% of the gut microbiota. The specific composition of bacteria population in each intervention is depicted in Supplementary Materials, Table S2. However, an extra portion of extrinsic wheat fiber in the form of food and drink did not affect alpha-diversity. Regarding the 259 OTUs investigated, relative abundance of OTU 64, *Blautia faecis* within the phylum *Lachnospiraceae*, was significantly increased in the intervention group compared to the placebo group (*p* = 0.04; only drink). No significant changes could be detected in the other group where extrinsic wheat fiber was induced in the form of food.

### 3.5. Analysis of Dietary Protocols

Total fiber intake increased by 13 ± 6 g/day in the food group and by 9 ± 6 g/day in the drink group, respectively. Participants receiving the extrinsic wheat fiber-enriched diet in form of solid food items had no significant changes regarding energy intake, carbohydrate, protein, and fat intake during the intervention period (Table 3).

### 3.6. Evaluation of Product Liking

Based on the same appearance and taste, participants could not distinguish between the control diet and the extrinsic wheat fiber-enriched products. After eating the respective foods, participants were asked to evaluate each product. All participants liked the products they had consumed and evaluated both of them equally (with and without extrinsic wheat fiber, average 8.2 of 10 points). However, extrinsic wheat fiber-enriched pizza was slightly, but significantly, rated worse than the un-supplemented equivalent. In contrast, extrinsic wheat fiber-enriched bread was significantly better evaluated. It is noteworthy that participants revealed no significant differences between all other products.

## 4. Discussion

The purpose of our study was, therefore, to assess the impact of a regular diet supplemented with extrinsic wheat fiber on fecal bulk (VITACEL^®^). We aimed to achieve an even distribution over the day together with the main meals administrating three times 3.34 g extrinsic wheat fiber. Extrinsic wheat fiber in food contains only pure wheat plant fiber that is composed of 76% cellulose and 24% hemicellulose. Moreover, our extrinsic wheat fiber product does not include starch residues, oils, and proteins. Furthermore, the absence of common bran products such as fructanes might also support beneficial effects for people with irritable bowel syndrome (IBS). More fiber used in those patients has been shown to be supportive [22,23]. Fecal bulk was significantly increased by the extrinsic wheat fiber-enriched diet in the form of solid foods given as muffins, rolls, pizza, etc. The increase of fecal wet weight is comparable with results from a recent study from the Netherlands reporting increased fecal wet weight in response to a 20 g extrinsic wheat fiber-enriched diet for 10 days [24]. Consistently, Jenkins et al. calculated a linear dose response per gram additional wheat fiber intake, resulting in a mean 2.7 g increase in fecal weight [25]. As we only observed significant changes in fecal wet weight, and not in the dry matter, we hypothesize that only the water content increased, but not the organic fraction in stool. It might also be that this moderate amount of fiber supplementation is not enough to detect significant changes in the dry material as Wit et al. was able to show with 20 g of fiber supplementation.

Furthermore, extrinsic wheat fiber-enrichment in foods did not affect the stool frequency per day. This finding might be attributed to the low subject number and the missing power that was only calculated according to fecal bulk. This does not contradict the results in the review of Vries et al. [13], which reports that each extra gram of extrinsic wheat fiber increases stool frequency by 0.004 ± 0.002 times/d (*p* = 0.0346). The published studies had 18 participants and more to calculate the stool frequency, which could explain the missing significance. Moreover, it is interesting to note, that mean stool consistency categorized according to the Bristol Stool Form Scale [17] did not change after five days of an extrinsic wheat fiber-enriched diet in form of foods. This finding is in line with data from Wit et al. [24] and shows that fiber supplementation is a highly tolerable intervention without major side effects like bloating or diarrhea.

Due to missing data, the second intervention was designed as a pilot study where we investigated whether additional fiber provided as a beverage resulted in an increased fecal wet weight as well. Fecal wet weight did not significantly increase in the group consuming extrinsic wheat fiber in form of drinks. Likewise, the other parameters like stool frequency per day and stool consistency did not change. In this context, the food matrix might play an important role for the physical and biochemical processes of digestion [26]. Wyman et al. investigated the effect of two doses of raw wheat bran (12 and 20 g/day) and two doses of cooked wheat bran (13.2 and 22 g/day) with the result that both doses of raw bran increased fecal dry weight, but only the higher dose decreased transit time and increased stool volume [27].

In our study, enrichment with 10 g of dietary fibers increased the fecal wet weight in cooked or baked products in contrast to fiber-enriched drinks. It seemed that baked or cooked extrinsic wheat fiber exhibited different effects on the gastrointestinal tract compared to raw, unprocessed extrinsic wheat fiber. It can also not be excluded that certain components of the (control) instant drink, which had to be included due to technical and taste sensory reasons, may have interfered with the results (Supplementary Materials, Tables S3 and S4.

Another important aspect of potentially beneficial effects of dietary fibers affects their metabolization to SCFAs. These are metabolites from those indigestible food components that are necessary for the alimentation of enterocytes. As humans are unable to digest fibers, we take advantage of the intestinal microbiota, which produces SCFAs like acetate or butyrate [28]. In the current study, we were not able to assess significant differences with regard to SCFAs measured in feces between the control and extrinsic wheat fiber-enriched diet, neither in the form of food nor drinks. In this context, it is yet unclear if fecal SCFAs concentrations resemble fiber intake at this low concentration due to the rapid absorption or metabolization of these metabolites. Therefore, differences in acetic acid—which was significantly elevated in the extrinsic wheat fiber-enriched diet in the form of food compared to drinks, and propionic was higher between control interventions—might have occurred by chance. The more so, as it was not intended to control the diet of our volunteers, which may have interfered with the results.

As microbiota composition is sensitive to external factors such as diet composition, we were interested in analyzing microbiota composition in response to fiber intake. However, in our study, the addition of extrinsic wheat fiber to the normal diet did not alter general alpha- and beta-diversity in both interventions, although existing studies indicating that rather short exposure times of a few days may be sufficient to induce changes of the microbiome [29,30]. Nevertheless, the extrinsic wheat fiber-enriched drinks induced a significant increase of *Blautia faecis* belonging to the family *Lachnospiraceae*. Other bacteria were not significantly altered in either group.

A strength of the present study was the high degree of standardization. The study was performed in a specialized study unit and participants had to eat their meals under supervision. The dietary intervention was carried out with standardized products. Moreover, fiber-enriched products and control products had nearly identical tastes and appearances. In addition, the study applied an array of different methods to investigate the impact of a regular diet supplemented with 10 g extrinsic wheat fiber on fecal bulk: fecal wet and dry weight, stool consistency, stool frequency, quantitation of SCFAs, and gut microbiota composition. As a limitation, we did not assess physical activity level of subjects over the study period, which might influence bowel movements and, therefore, stool frequency. Due to missing differences between the groups, we assume this influence as negligible. Secondly, the different composition between food and beverage. Lower effectivity in fecal bulk might be relaxed to the lower content of cellulose in the beverage. Thirdly, for preventive aspects, we recruited volunteers of a younger age. Therefore, our results on fecal bulk might not be comparable to a senior with restricted drinking habits.

## 5. Conclusions

In conclusion, extrinsic wheat fiber supplementation is a convenient and well-tolerated possibility to significantly increase total fiber intake by at least 10 g/day. Our results suggest that the food matrix in which fiber supplements are administered might play an important role for fecal bulk, as a substantial increase was only detectable with solid food items under our conditions.

## Figures and Tables

**Table 1:** Impact of added extrinsic wheat fiber intake on fecal bulk.

	Food			Drink		
	Control Diet	Extrinsic Wheat Fiber-Enriched Diet	*p*-Value	Control Diet	Extrinsic Wheat Fiber-Enriched Diet	*p*-Value
*n*	10 (5 ♀,5 ♂)			19 (12 ♀,7 ♂)		
Fecal Wet Weight (gram per day)	144.4 ± 38.1	209.1 ± 88.0	0.02	171.2 ± 89.5	176.1 ± 64.9	0.51
Fecal Dry Weight (gram per day)	36.3 ± 16.4	33.7 ± 17.2	0.56	40.5 ± 31.3	41.8 ± 18.7	0.18
Stool Consistency ^a^	3.5 ± 0.8	3.9 ± 1.2	0.53	3.6 ± 0.7	3.5 ± 0.7	0.72
Stool Frequency (per day)	1.2 ± 0.4	1.3 ± 0.5	0.40	1.2 ± 0.32	1.2 ± 0.3	0.78

Data are presented as mean ± standard deviation. *p*-value < 0.05 was regarded as statistically significant. According to distribution, either the paired *t*-test or Wilcoxon-signed ranked test was applied to assess differences between control diet and extrinsic wheat fiber-enriched diet. ns, not significant. a, categorized according to Bristol Stool Form Scale [17].

**Table 2:** SCFA concentrations measured in fecal samples.

	Food			Drink		
	Control Diet	Extrinsic Wheat Fiber-Enriched Diet	*p*-Value	Control Diet	Extrinsic Wheat Fiber-Enriched Diet	*p*-Value
*n*	10 (5 ♀,5 ♂)			19 (12 ♀,7 ♂)		
Acetic Acid (ng/mL)	2633.3 ± 981.5	3131.9 ± 1300.9 ^a^	0.10	2420.2 ± 1298.3	2038.7 ± 1208.7 ^a^	0.26
Butyric Acid (ng/mL)	1187.3 ± 688.0	1102.3 ± 645.8	0.72	1252.8 ± 650.2	1041.8 ± 593.0	0.13
Propionic Acid (ng/mL)	736.4 ± 259.3 ^b^	771.4 ± 209.0	0.65	1087.6 ± 469.9 ^b^	857.5 ± 317.2	0.10
2-Methylbutyric Acid (ng/mL)	72.6 ± 62.1	81.0 ± 41.2	0.98	77.5 ± 73.8	73.0 ± 43.3	0.77
Hexanoic Acid (ng/mL)	151.0 ± 121.0	162.8 ± 145.6	0.75	115.5 ± 137.7	112.2 ± 127.1	0.47
Isobutyrate (ng/mL)	347.2 ± 493.9	234.1 ± 419.0	0.49	105.6 ± 76.2	101.6 ± 49.1	0.80
Isovalerate (ng/mL)	98.8 ± 73.4	91.3 ± 52.7	0.79	92.6 ± 82.1	87.0 ± 46.2	0.75
Pentanoic Acid (ng/mL)	181.2 ± 67.7	170.8 ± 71.9	0.61	194.3 ± 91.2	154.3 ± 55.1	0.10

Data are presented as mean ± standard deviation. *p*-value < 0.05 was regarded as statistically significant. According to normality distribution, either the paired *t*-test or Wilcoxon-signed ranked test was applied to assess differences between control and the extrinsic wheat fiber-enriched diets. ns, not significant. a = significances between extrinsic wheat fiber-enriched diets; b = differences between control diets.

**Table 3:** Macronutrient intake.

	Food			Drink		
	Control Diet	Extrinsic Wheat Fiber-Enriched Diet	*p*-Value	Control Diet	Extrinsic Wheat Fiber-Enriched Diet	*p*-Value
*n*	10 (5 ♀,5 ♂)			19 (12 ♀,7 ♂)		
Energy Intake (kcal per day	2535 ± 515	2637 ± 321	0.41	2097 ± 474	2299 ± 521	0.17
Carbohydrates (gram per day)	289 ± 92 (47 EN%)	284 ± 48 (44 EN%)	0.98	232 ± 45 (45 EN%)	257 ± 50 (45 EN%)	0.13
Protein (gram per day)	85 ± 15 (13 EN%)	90 ± 17 (14 EN%)	0.22	83 ± 32 (16 EN%)	90 ± 36 (16 EN%)	0.56
Fat (gram per day)	109 ± 22 (40 EN%)	117 ± 20 (42 EN%)	0.15	85 ± 32 (37 EN%)	91 ± 33 (37 EN%)	0.33
Fiber Total (gram per day)	22 ± 7	35 ± 6	<0.0001	25 ± 10	35 ± 10	<0.01

Data are presented as mean ± standard deviation. *p*-value < 0.05 was regarded as statistically significant. According to normality distribution, either the paired t-test or Wilcoxon-signed ranked test was applied to assess differences between control diet and extrinsic wheat fiber-enriched diet. EN %, energy percent; ns, not significant.

## Supplementary Materials

The following are available online at https://www.mdpi.com/2072-6643/12/2/298/s1, Figure S1: Fiber-enriched products (M.Z) and the control products (Y.N), Methods S2: Quantification of short-chain fatty acids (SCFA), Table S3: Anthropometric and body composition characteristics in both intervention phases at baseline, Table S4: The specific composition of bacteria population, Table S5: Ingredients of supplemented food and drink.
