# Supplementary material for: Effects of Extrinsic Wheat Fiber Supplementation on Fecal Weight; A Randomized Controlled Trial
Source: Nutrients. 2020 Jan 22;12(2):298. doi: 10.3390/nu12020298 (PMC7070730; doi:10.3390/nu12020298)
Supplement: Supplementary file 1 [file nutrients-12-00298-s001.zip › Brandl et al_S1_Supplementary Material_Figure 1.docx]

**Suppplementary material, Figure 1**

S1: Fiber enriched products (M,Z) and the control products (Y,N)

**A B C D E**
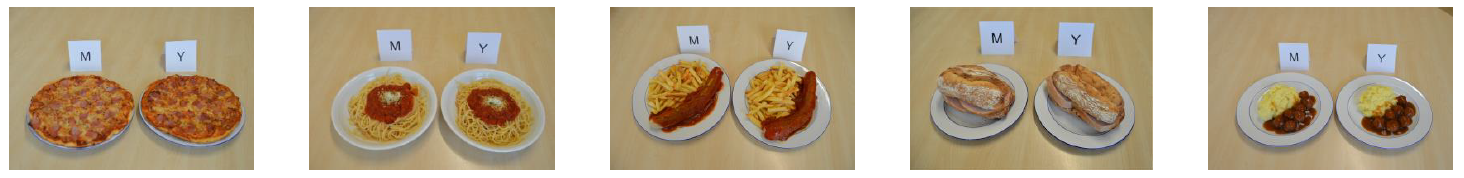


**F G H I J**
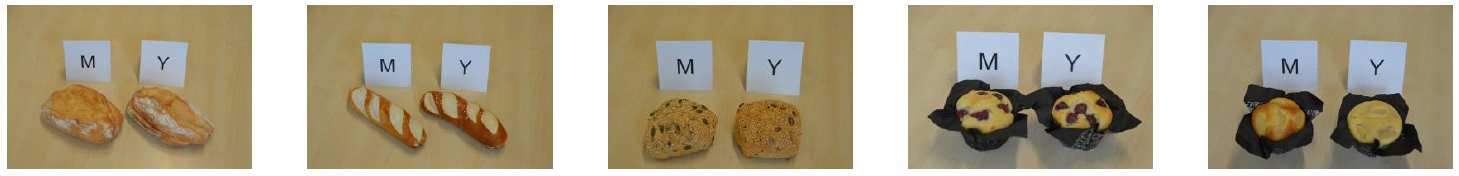


**K L**


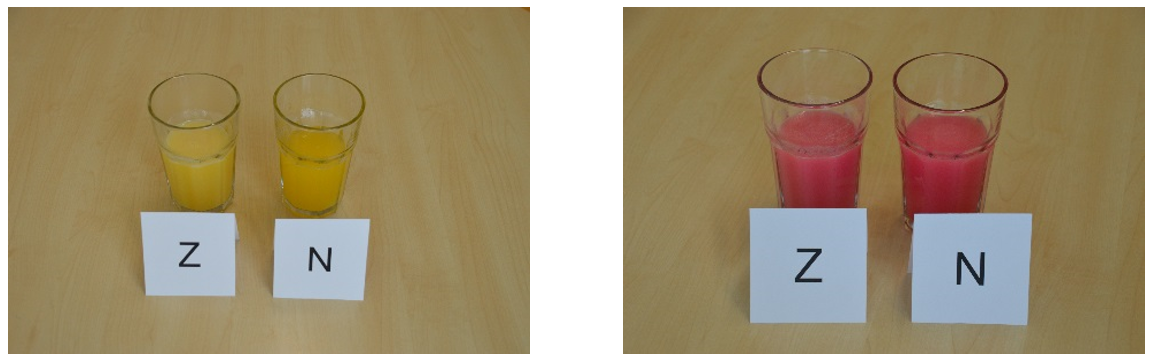


**Figure 1.** A, pizza; B, spaghetti with tomato sauce; C, curry sausage; D, meat loaf; E, meat balls with mashed potatoes; F, roll; G, pretzel breadstick; H, whole grain rolls; I, muffin with cherry; muffin with pear; K; drink flavored with peach and passion fruit; L, drink flavored with cherry
