# Supplementary material for: Effects of Extrinsic Wheat Fiber Supplementation on Fecal Weight; A Randomized Controlled Trial
Source: Nutrients. 2020 Jan 22;12(2):298. doi: 10.3390/nu12020298 (PMC7070730; doi:10.3390/nu12020298)
Supplement: Supplementary file 1 [file nutrients-12-00298-s001.zip › Brandl et al_S2_Supplementary Material.docx]

**Supplemental material**

S2: Quantitation of short-chain fatty acids (SCFAs)

SCFA measurement was performed by LC-MS/MS after 3-nitrophenylhydrazine derivatization using a recently reported method with some modifications (Han et al. 2015). Frozen fecal samples (100 mg) were precisely weighed, suspended in 5 ml of an internal standard solution containing propionic acid-d5, ^13^C_2_-acetate and ^13^C_4_-butyrate in acetonitrile/water (1 + 1, *v*/*v*, 5 ml), and homogenized by vortexing in a Precellys homogenizer evolution (Bertin Technologie, Montigny-le-Bretonneux, France, Tubes CK28_15 ml, ceramic beads diameter 2,8 mm, 6300 rpm, 3 x 20 s with 20 s breaks). After equilibration (30 min) on an orbital shaker, samples were centrifuged (4000 rpm, 6 °C), and supernatants (40 μl) were placed into autosampler vials, mixed with 20 μl of 3-nitrophenylhydrazine hydrochloride (200 mmol/l) in acetonitrile/water (1/1, *v*/*v*) and 20 μl of N-(3-dimethylaminopropyl)-N′-ethylcarbodiimide hydrochloride (120 mmol/L) in acetonitrile/water (1/1, *v*/*v*) containing 6% pyridine. After 30 min at 40 °C, samples were diluted with acetonitrile/water (1/1, *v*/*v*; 920 μl) and aliquots (1 μl) were used for UHPLC-MS/MS analysis.

A Exion UHPLC system (Sciex, Darmstadt, Germany), consisting of two LC pumps LC30AD, a DGU-20 degasser, a SIL-30AC autosampler, a CTO-30A column oven, and a CBM-20A system controller, was hyphenated with a QTRAP 6500+ LC-MS/MS system (Sciex, Darmstadt, Germany). Chromatographic separation was performed on a Kinetex C18 column (100 × 2.1 mm, 1.7 μm, 100 Ǻ, Phenomenex, Aschaffenburg, Germany) using water/formic acid (100/0.1, v/v) as solvent A and acetonitrile/formic acid (100/0.1, v/v) as solvent B at a flow rate of 0.4 ml/min and a column temperature of 40 °C. Starting with initial conditions of 23% B for 3 min, the content of B in the mobile phase was increased to 30% within 4 min, and to 5,5 min increased to 40% of solvent B, followed by an immediate switch to 100% B (held for 1 min), and re-equilibration at starting conditions for 3 min.

The mass spectrometer was operated in the negative electrospray ionization and low mass mode, and the ion spray voltage was set at − 4500 V. Nebulizer gas (55 psi), turbo gas (500 °C) for solvent drying (65 psi), curtain gas (35 psi), and collision gas (1.9 × 10^− 5^ Torr). The MS/MS parameters, declustering potential, entrance potential, collision cell entrance potential, collision energy, and cell exit potential were tuned for each individual compound after derivatization by flow injection (10 μl/min), detecting the fragmentation of the [M-H]-molecular ions into specific product ions after collision with nitrogen (4.5 × 10^− 5^ Torr). Mass spectrometric data were analyzed using Analyst software 1.6.3 (Sciex). Target analytes were detected based on scheduled MRM mode using the following mass transitions: acetate (m/z 193.9 → 136.8), propanoate (m/z 207.9 → 136.8), butyrate (m/z 221.9 → 136.9), isobutyrate (m/z 222.0 → 136.9), valerate (m/z 236.0 → 136.8), isovalerate (m/z 236.0 → 137.0), 2-methylbutyrate (m/z 236.0 → 136.8), hexanoate (m/z 250.0 → 136.7), and methylvalerate (m/z 250.0 → 136.9). While acetate and propanoate were quantified using their isotopologues ^13^C_2_-acetate (m/z 196.0 → 136.9) and d5-propionate (m/z 213.0 → 136.9), the remaining SCFAs were determined using ^13^C_4_-butyrate (m/z 226.0 → 137.0) as internal standard. After UHPLC-MS/MS analysis, calibration curves (1,37640–1393,4 mg/l; eleven-point calibration) were prepared by plotting peak area ratios of analyte to internal standard against concentration ratios of each analyte to the internal standard using linear regression (R_2_ > 0.996). For each sample, data were calculated as the means of triplicate analysis.
