# Supplementary material for: Effects of Extrinsic Wheat Fiber Supplementation on Fecal Weight; A Randomized Controlled Trial
Source: Nutrients. 2020 Jan 22;12(2):298. doi: 10.3390/nu12020298 (PMC7070730; doi:10.3390/nu12020298)
Supplement: Supplementary file 1 [file nutrients-12-00298-s001.zip › Revised_Brandl et al_S3_Supplementary Material_Table 1.docx]

**Supplemental material, Table 1**

S3: Anthropometric and body composition characteristics in both intervention phases at baseline

|  | **Food** | | | Drink | | |
| --- | --- | --- | --- | --- | --- | --- |
|  | **Control Diet** | **Extrinsic Wheat Fiber-Enriched Diet** | ***p*-Value** | Control Diet | Extrinsic Wheat Fiber-Enriched Diet | *p*-Value |
| *n* | 10 (5 ♀, 5 ♂) | |  | 19 (12 ♀, 7 ♂) | |  |
| Age (years) | 25 ± 4 | |  | 24 ± 4 | |  |
| Waist circumference (cm) | 74.3 ± 5.7 | 74.3 ± 5.9 | 0.89 | 73.3 ± 7.4 | 73.4 ± 7.4 | 0.96 |
| Body weight (kg) | 69.2 ± 8.7 | 69.9 ± 9.7 | 0.21 | 66.7 ± 10.9 | 66.5 ± 10.9 | 0.94 |
| Body mass index (kg/m²) | 22.4 ± 1.9 | 22.6 ± 2.2 | 0.22 | 22.0 ± 2.0 | 22.0 ± 2.0 | 0.89 |
| Fat free mass (kg) | 54.8 ± 11.6 | 55.6 ± 12.0 | 0.06 | 52.0 ± 10.8 | 51.9 ± 11.2 | 0.98 |
| Fat mass (kg) | 14.4 ± 4.9 | 14.3 ± 4.5 | 0.66 | 14.7 ± 3.9 | 14.6 ± 3.9 | 0.99 |

Data are presented as mean ± standard deviation. *p*-values <0.05 were regarded as statistically significant. According to distribution either paired t-test or Wilcoxon-signed ranked test was applied to assess differences between control diet and extrinsic wheat fiber-enriched diet. ns, not significant.
