# Supplementary material for: Effects of Extrinsic Wheat Fiber Supplementation on Fecal Weight; A Randomized Controlled Trial
Source: Nutrients. 2020 Jan 22;12(2):298. doi: 10.3390/nu12020298 (PMC7070730; doi:10.3390/nu12020298)
Supplement: Supplementary file 1 [file nutrients-12-00298-s001.zip › Revised_Brandl et al_S4_Supplemental Material_Table 2.docx]

**Supplemental material, Table 2**

S4: The specific composition of bacteria population

|  | **Food** | | | Drink | | |
| --- | --- | --- | --- | --- | --- | --- |
|  | **Control Diet** | **Extrinsic Wheat Fiber-Enriched Diet** |  | Control Diet | Extrinsic Wheat Fiber-Enriched Diet |  |
| *n* | 10 (5 ♀, 5 ♂) | |  | 19 (12♀, 7 ♂) | |  |
| *Firmicutes,* % of gut microbiota | 64.51 | 65.74 |  | 52.16 | 58.15 |  |
| *Bacteroidetes,* % of gut microbiota | 32.47 | 31.18 |  | 43.74 | 38.93 |  |
| *Proteobacteria,* % of gut microbiota | 1.49 | 1.12 |  | 2.57 | 1.90 |  |
| *Actinobacteria,* % of gut microbiota | 1.36 | 1.38 |  | 1.03 | 0.53 |  |
| *Verrucomicrobia,* % of gut microbiota | 0.11 | 0.19 |  | 0.22 | 0.35 |  |
| *Unkown Bacteria,* % of gut microbiota | 0.06 | 0.39 |  | 1.31 | 0.67 |  |
