# Supplementary material for: Effects of Extrinsic Wheat Fiber Supplementation on Fecal Weight; A Randomized Controlled Trial
Source: Nutrients. 2020 Jan 22;12(2):298. doi: 10.3390/nu12020298 (PMC7070730; doi:10.3390/nu12020298)
Supplement: Supplementary file 1 [file nutrients-12-00298-s001.zip › Revised_Brandl et al_S5_Supplementary Material_Table 3,4.docx]

**Supplementary material, Table 3,4**

S5: Ingredients of supplemented food and drink

**Food - extrinsic wheat fiber**

3.34 g extrinsic wheat fiber in each product. 3 Products were eaten by every participant per day.

**Food- placebo**

The same food were produced but without wheat fiber

**Table 3: Drink - Powder mix – extrinsic wheat fiber- enriched diet**

| **Component** | **Cherry (%)** | **Peach-Passion fruit (%)** |
| --- | --- | --- |
| VITACEL wheat plant fibre | 45.00 | 45.00 |
| VITACEL psyllium | 3.75 | 3.75 |
| Guar | 3.75 | 3.75 |
| Isomaltulose | 42.00 | 42.65 |
| Sucralose | 0.125 | 0.100 |
| Citric acid | 4.13 | 3.13 |
| Flavour cherry | 0.875 | 0.00 |
| Flavour peach-passion fruit | 0.00 | 1.25 |
| Ponceau 4R E 124 | 0.38 | 0.38 |

**Table 4: Drink - Powder mix – control diet**

| **Component** | **Cherry (%)** | **Peach-Passion fruit (%)** |
| --- | --- | --- |
| VITACEL powdered celluose | 3.75 | 3.75 |
| VITACEL psyllium | 3.75 | 3.75 |
| Guar | 3.75 | 3.75 |
| Isomaltulose | 50.75 | 51.78 |
| Sucralose | 0.125 | 0.100 |
| Citric acid | 4.125 | 2.75 |
| Flavour cherry | 0.875 | 0.000 |
| Flavour peach-passion fruit | 0.000 | 1.250 |
| Ponceau 4R E 124 | 0.375 | 0.375 |
| Modified corn starch E 1442 | 25.00 | 25.00 |
| Potato flakes | 7.50 | 7.50 |
